# Synthesis and characterisation of 5-acyl-6,7-dihydrothieno[3,2-*c*]pyridine inhibitors of Hedgehog acyltransferase

**DOI:** 10.1016/j.dib.2016.02.012

**Published:** 2016-02-10

**Authors:** Thomas Lanyon-Hogg, Naoko Masumoto, George Bodakh, Antonio D. Konitsiotis, Emmanuelle Thinon, Ursula R. Rodgers, Raymond J. Owens, Anthony I. Magee, Edward W. Tate

**Affiliations:** a Department of Chemistry, Imperial College London, SW7 2AZ, UK; Institute of Chemical Biology, Imperial College London, SW72AZ, UK; b Molecular Medicine Section, National Heart & Lung Institute, Imperial College London, London SW7 2AZ, UK; c Oxford Protein Production Facility UK, The Research Complex at Harwell, Rutherford Appleton Laboratory, Harwell Science & Innovation Centre, Harwell OX11 0FA, UK

**Keywords:** Synthesis, Inhibitors, Hedgehog acyltransferase, Conformation

## Abstract

In this data article we describe synthetic and characterisation data for four members of the 5-acyl-6,7-dihydrothieno[3,2-*c*]pyridine (termed “RU-SKI”) class of inhibitors of Hedgehog acyltransferase, including associated NMR spectra for final compounds. RU-SKI compounds were selected for synthesis based on their published high potencies against the enzyme target. RU-SKI 41 (**9a**), RU-SKI 43 (**9b**), RU-SKI 101 (**9c**), and RU-SKI 201 (**9d**) were profiled for activity in the related article “Click chemistry armed enzyme linked immunosorbent assay to measure palmitoylation by Hedgehog acyltransferase” (Lanyon-Hogg et al., 2015) [1]. ^1^H NMR spectral data indicate different amide conformational ratios between the RU-SKI inhibitors, as has been observed in other 5-acyl-6,7-dihydrothieno[3,2-*c*]pyridines. The synthetic and characterisation data supplied in the current article provide validated access to the class of RU-SKI inhibitors.

## **Specification table**

**Table:** 

Subject area	*Chemistry*
More specific subject area	*Organic Synthesis*
Type of data	*Synthetic scheme, experimental procedures, physical data, NMR spectra*
How data was acquired	NMR (Bruker AM-400 or AM-500). High resolution mass spectrometry (AUTOSPEC P673 spectrometer). *Microwave reactions (Biotage Initiator)*
Data format	*Analysed*
Experimental factors	*N/A*
Experimental features	*Synthesis performed using standard organic chemistry techniques without inert atmosphere, unless otherwise stated.*
Data source location	*N/A*
Data accessibility	*Data are available in this article*

## **Value of the data**

• Validated synthetic route to substituted 5-acyl-6,7-dihydrothieno[3,2-*c*]pyridines.
• The synthesised compounds can be used as inhibitors of Hedgehog acyltransferase (Hhat), termed “RU-SKI” inhibitors.
• Synthetic data provides route for development of other Hhat inhibitors based on this molecular core with improved activity profiles.
• NMR spectral data demonstrate biologically active RU-SKI compounds possess variable amide conformational preferences, which can be modulated.

## 1 Data

This article describes the synthesis and characterisation of four 5-acyl-6,7-dihydrothieno[3,2-*c*]pyridine (“RU-SKI”) inhibitors of Hedgehog acyltransferase (Hhat) which were employed in dose–response analysis in the related article “Click-chemistry armed enzyme linked immunosorbent assay to measure palmitoylation by Hedgehog acyltransferase” [1]. The RU-SKI inhibitors were identified and developed by Resh and co-workers [2], [3], and the compounds with the highest published potencies against Hhat were selected for synthesis. RU-SKI 41 (**9a**), RU-SKI 43 (**9b**), RU-SKI 101 (**9c**) and RU-SKI 201 (**9d**) were synthesised according to our previously reported synthetic strategy to access the 5-acyl-6,7-dihydrothieno[3,2-*c*]pyridine core scaffold [4]. Inhibitors were analysed in our Click Chemistry Armed Enzyme Linked Immunosorbent Assay, displaying low- and sub-micromolar IC_50_ values against Hhat [1].

**Fig. 1:**
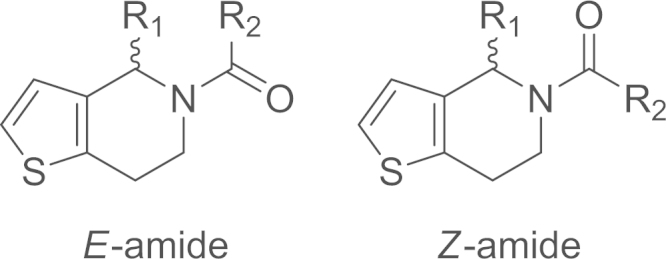
*E*- and *Z*-amide conformations adopted by the 5-acyl-6,7-dihydrothieno[3,2-*c*]pyridine core of the RU-SKI compounds.

**Fig. 2:**
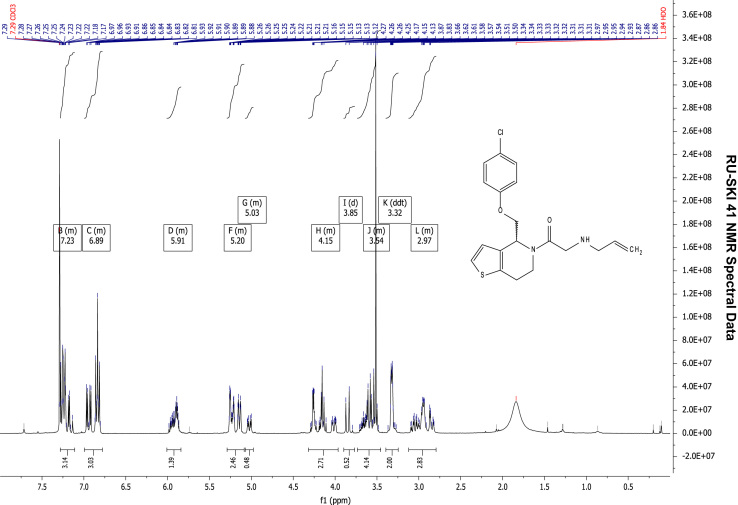
^1^H NMR (400 MHz, CDCl_3_) of RU-SKI 41 (**9a**).

As demonstrated in our previous study of the 5-acyl-6,7-dihydrothieno[3,2-*c*]pyridine core [4], the amide in the RU-SKI compounds also adopts two conformations (Fig. 1). The conformational preference is affected by non-covalent interactions between the amide carbonyl and neighbouring substituents [4]. Altered conformational ratios are observed in the ^1^H NMR data of the RU-SKI compounds (Table 1, Fig. 2, Fig. 6, Fig. 10, Fig. 14). The synthetic, characterisation and conformational data of compounds **9a**–**9d** is reported here, along with NMR spectra of final RU-SKI inhibitors.

## 2 Experimental design, materials and methods

### 2.1 Materials

Materials and equipment were as previously described [4].

### 2.2 Abbreviations

EDC (1-ethyl-3-(3-dimethylaminopropyl)carbodiimide), PyBOP ((benzotriazol-1-yloxy)tripyrrolidinophosphonium hexafluorophosphate), TEA (triethylamine), DIPEA (N,N-diisopropylethylamine), DMF (dimethylformamide), DCM (dichloromethane), HOBt (hydroxybenzotriazole), TFA (trifluoroacetic acid), chex (cyclohexane).

### 2.3 General procedures

**Scheme 1:**
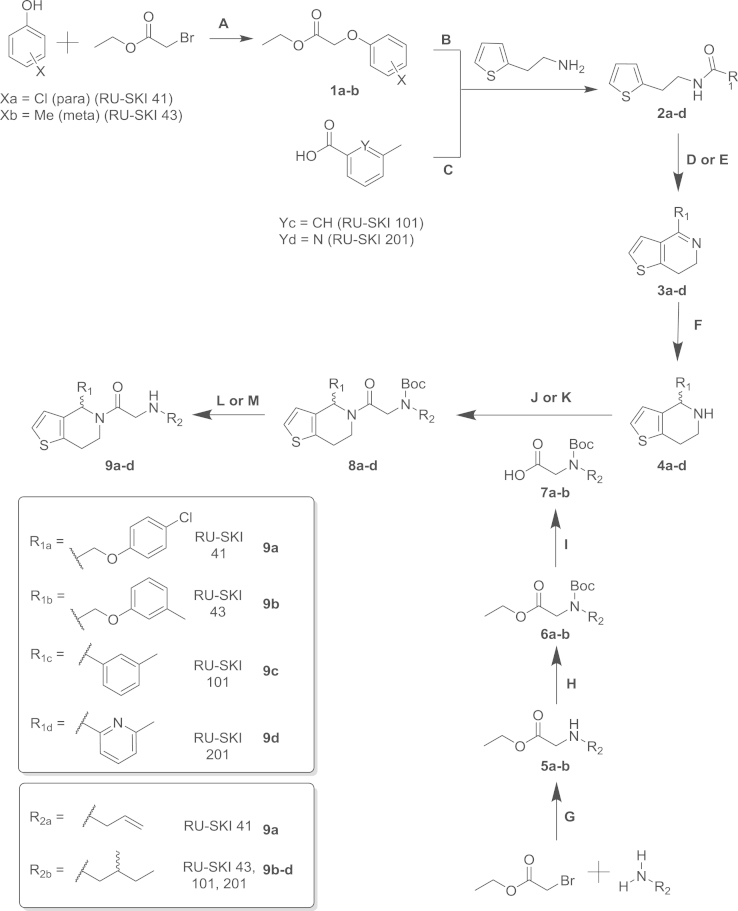
General procedures. (A) K_2_CO_3_, acetone, room temperature, overnight. (B) NaOMe, MeOH, room temperature, overnight. (C) PyBOP, DIPEA, DMF, room temperature, overnight. (D) POCl_3_, P_2_O_5_, toluene, 140 °C, microwave, 30 min. (E) POCl_3_, P_2_O_5_, xylene, 85 °C, 2 h. (F) NaBH_4_, MeOH, room temperature, 1 h or overnight. G. DCM, 0 °C to room temperature, overnight. (H) TEA, Boc_2_O, MeOH, 0 °C to 60 °C, 1 h. (I) 1 M LiBH_4_ in H_2_O, THF, room temperature, 1 h. (J) PyBOP, DIPEA, DCM, room temperature, overnight. (K) EDC, HOBt, DMF, room temperature, overnight. (L) TFA: DCM (1:1), room temperature. (M) 4 M HCl in dioxane, room temperature.

Synthesis of RU-SKI 41, 43, 101 and 201 followed our previously reported synthetic strategy (Scheme 1) [4].

### 2.3.1 General procedure A (ethyl phenoxy acetate preparation)

2-Bromoethylacetate (0.66 mL, 5.99 mmol, 1 eq) was added dropwise to a solution of K_2_CO_3_ (1660 mg, 12.0 mmol, 2 eq) and phenol (5.99 mmol, 1 eq) in acetone (25 mL) and stirred at room temperature overnight. The reaction mixture was concentrated *in vacuo* then dissolved in brine and extracted with ethyl acetate. The combined organic layers were dried over Na_2_SO_4_ and the solvent removed *in vacuo*. The phenyl ether was used without further purification.

### 2.3.2 General procedure B (thiophene ethylamide preparation using sodium methoxide)

Phenoxyethyl acetate (3.91 mmol, 1 eq) was dissolved in methanol (20 mL), and sodium methoxide solution (0.5 M, 2.12 mL, 19.5 mmol, 5 eq) added dropwise to the reaction mixture. 2-(3-Thienyl)ethylamine (0.47 mL, 508 mg, 3.91 mmol, 1 eq) was added dropwise and stirred overnight at room temperature. The solvent was removed *in vacuo* and the resulting crude material was dissolved in brine, extracted with ethyl acetate and the combined organic layers washed with water. The organic layer was dried over Na_2_SO_4_, concentrated *in vacuo* and the crude residue purified by flash column chromatography.

### 2.3.3 General procedures C, D, E, F, and N

General procedures C, D, E, F, and N were performed as previously described [4].

### 2.3.4 General procedure G

General procedure G was performed as previously described [5].

### 2.3.5 General procedure H

General procedure G was performed as previously described [6].

### 2.3.6 General procedure I (ester hydrolysis of ethyl aminoacetate/preparation of ethyl amino acetic acid)

Boc-protected ethyl aminoacetate (0.15 mmol, 1 eq) was dissolved in THF (5 mL), lithium hydroxide (1 M solution, 0.4 mL, 0.40 mmol, 3.8 eq) added and the reaction stirred overnight at room temperature. If necessary, more lithium hydroxide was added to the reaction mixture in order to drive the reaction to completion. The reaction was acidified with concentrated hydrochloric acid (pH 2) and extracted in ethyl acetate. The combined organic layers were dried over Na_2_SO_4_, concentrated *in vacuo*, and purified by column chromatography or used without further purification.

### 2.3.7 General procedure J (coupling of the side chain using PyBOP) (RU-SKI 41/43)

The amine obtained from general procedure F (0.11 mmol, 1 eq) was added to a solution of the acid obtained from general procedure I (0.12 mmol, 1.1 eq), DIPEA (52 μL, 0.30 mmol, 2.75 eq) and PyBOP (56 mg, 0.11 mmol, 1 eq) in DCM (5 mL) and the reaction stirred overnight at room temperature. The reaction was quenched by addition of water and extracted in ethyl acetate. The combined organic layers were washed with water and brine, dried over Na_2_SO_4,_ concentrated *in vacuo*, and purified by flash column chromatography.

### 2.3.8 General procedure K (coupling of the side chain using EDC/HOBt) (RU-SKI 101/201)

The amine obtained from general procedure F (0.043 mmol, 1 eq) and the Boc-protected acetic acid obtained from general procedure I (0.043 mmol, 1 eq) were dissolved in DMF (~2 mL). HOBt (5.8 mg, 0.043 mmol, 1 eq), DIPEA (15 μL, 0.086 mmol, 2 eq) and EDC (12.4 mg, 0.065 mmol, 1.5 eq) were added to the reaction mixture and the reaction stirred overnight at room temperature. DCM was added and the solution washed with aqueous LiCl (5% w/w) and brine. The organic layer was dried over Na_2_SO_4_, concentrated *in vacuo*, and purified by flash column chromatography.

### 2.3.9 General procedure L (Boc deprotection by TFA)

The amide obtained from general procedure J or K (0.049 mmol, 1 eq) was dissolved in 1:1 mixture of DCM and TFA (5 mL). Afterwards, the solvent was removed *in vacuo* and the residual was neutralised by saturated sodium hydrogen carbonate, extracted with DCM three times and dried over MgSO_4_. The required amine was isolated using strong cation exchange resin and eluted with ammonia (2 M) in methanol to recover the free amine, and purified by flash column chromatography.

### 2.3.10 General procedure M (Boc deprotection by HCl in dioxane)

The amide obtained from general procedure J or K (0.18 mmol, 1 eq) was stirred in 4 M HCl-Dioxane (~5 mL) for 2 h at room temperature. The solvent was removed *in vacuo* and the residue dissolved in ethyl acetate, washed with water and brine and dried over MgSO_4_. The required amine was isolated using strong cation exchange resin and eluted with ammonia (2 M) in methanol to recover the free amine, and purified by flash column chromatography.

### 2.3.11 General procedure N (coupling of the side chain using acid chlorides)

The amine obtained from general procedure F (0.09 mmol, 1 eq) and TEA (25 μL, 18 mg, 0.18 mmol, 2 eq) were dissolved in dry DCM (1 mL). The corresponding acid chloride (0.11 mmol, 1.2 eq) was added and the reaction mixture stirred at room temperature for 2 h. The solvent was removed *in vacuo,* and the residue purified by flash column chromatography.

## 2.4 RU-SKI synthetic data

### 2.4.1 RU-SKI 41 synthetic data

#### 2.4.1.1 Ethyl (*p*-chlorophenoxy)acetate (**1a**)

**Figure:**
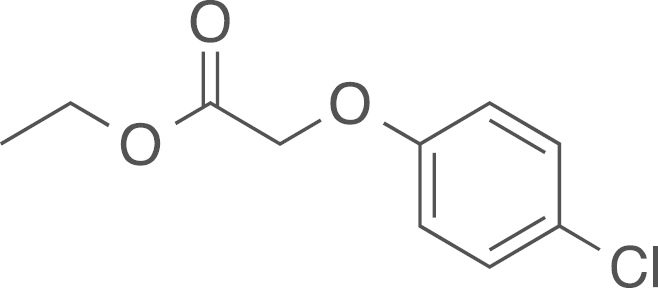

The ethyl (*p*-chlorophenoxy)acetate (**1a**) was obtained from 4-chlorophenol (0.59 mL, 770 mg, 5.99 mmol, 1 eq) and 2-bromoethylacetate (1 g, 5.99 mmol, 1 eq) using general procedure A as a white solid (1.28 g, 5.87 mmol, 98%). ^1^H NMR (400 MHz, CDCl_3_) δ=7.31–7.22 (m, 2H), 6.91–6.82 (m, 2H), 4.61 (s, 2H), 4.29 (q, ^*3*^*J*=7.1 Hz, 2H), 1.31 (t, ^*3*^*J*=7.1 Hz, 3H); ^13^C NMR (101 MHz, CDCl_3_) δ=168.61, 156.46, 129.46, 126.70, 116.02, 65.63, 61.48, 14.15; IR υ_MAX_ (neat)/cm^−1^: 2986 (CH_3_, -CH_2_-, alkyl), 1755.14 (C=O stretch, ester), 1595.32, 1584.14, 1489.92, 1441.44, 1379.18, 1292.86, 1192.13, 1171.21, 1075.91, 1026.29, 1006.67, 929.80, 872.46, 719.30; HRMS (ESI, *m/z*) calcd. for C_10_H_15_NO_3_Cl^+^ [M+NH_4_]^+^, 232.0740; found, 232.0738 [M+NH_4_]^+^.

#### 2.4.1.2 2-(*p*-Chlorophenoxy)-1-[2-(2-thienyl)ethylamino]-1-ethanone (2a)

**Figure:**
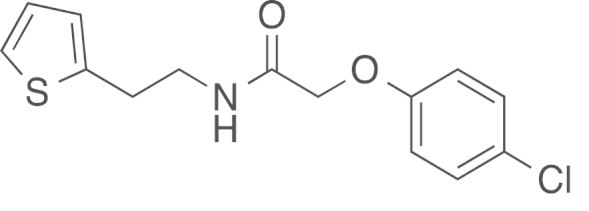

The amide (**2a**) was obtained from 2-(3-thienyl)ethylamine (0.47 mL, 500 mg, 3.91 mmol, 1 eq) and ethyl (4-chlorophenoxy) acetate (850 mg, 3.91 mmol, 1 eq) using general procedure B as a white solid (975 mg, 3.30 mmol, 73%). ^1^H NMR (400 MHz, CDCl_3_) δ=7.31–7.26 (m, 2 H), 7.19 (dd, ^*3*^*J*=5.1, ^*4*^*J*=1.1 Hz, 1 H), 6.96 (dd, ^*3*^*J*=5.1, 3.4 Hz, 1 H), 6.87–6.79 (m, 3 H), 4.48 (s, 2 H), 3.65 (q, ^*3*^*J*=6.5 Hz, 2 H), 3.10 (t, ^*3*^*J*=6.7 Hz, 2 H); ^13^C NMR (101 MHz, CDCl_3_) δ=167.79, 155.72, 140.71, 129.69, 127.09, 125.51, 124.14, 115.93, 67.60, 40.28, 29.83. IR υ_MAX_ (neat)/cm^−1^: 3408.37, 3302.15, 3093.46, 3064.49, 2939.45, 2921.72, 2856.33 (CH_3_, -CH_2_-, alkyl), 1654.90 (C=O stretch, amide), 1597.10, 1537.80, 1488.72, 1427.34, 1342.82, 1278.53, 1230.48, 1171.54, 1096.68, 1046.50, 1007.56, 826.76, 698.54, HRMS (ESI, *m/z*) calcd. for C_14_H_15_NO_2_SCl^+^ [M+H]^+^, 296.0512; found, 296.0507 [M+H]^+^.

#### 2.4.1.3 (*p*-Chlorophenoxy)(1-thia-5-aza-4,5,6,7-tetrahydroinden-4-yl)methane (4a) (ring closured imine 3a)/(amine 4a)

**Figure:**
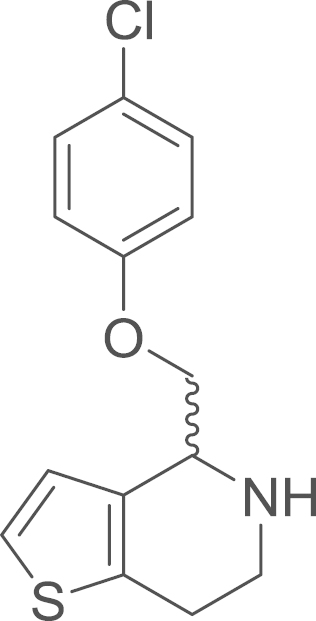

The cyclic imine (**3a**) was obtained from 2-(4-chlorophenoxy)-1-[2-(2-thienyl)ethylamino]-1-ethanone (**2a**) (348 mg, 1.18 mmol, 1 eq) using general procedure E as a brown oil (327 mg, 1.18 mmol, 100%). The crude material was used without further purification. ^1^H NMR (400 MHz, CDCl_3_) δ=δ 7.18–7.11 (m, 3 H), 7.04–6.99 (m, 3 H), 5.56 (s, 2 H), 4.11 (t, ^*3*^*J*=8.7 Hz, 2 H), 3.30 (t, ^*3*^*J*=8.7 Hz, 2 H). The amine (**4a**) was obtained from (4-chlorophenoxy)(1-thia-5-aza-6,7-dihydroinden-4-yl)methane (**3a**) (327 mg, 1.18 mmol, 1 eq) using general procedure F as yellow oil (181 mg, 0.65 mmol, 55%). ^1^H NMR (400 MHz, CDCl_3_) δ=7.28–7.23 (m, 2 H), 7.15 (d, ^*3*^*J*=5.2 Hz, 1 H), 6.91–6.87 (m, 3 H), 4.42–4.37 (m, 1 H), 4.22 (dd, ^*2*^*J*=9.1, ^*3*^*J*=3.8 Hz, 1 H), 4.05 (t, ^*3*^*J*=8.8 Hz, 1 H), 3.34 (dt, ^*2*^*J*=12.0, ^*3*^*J*=5.1 Hz, 1 H), 3.12 (ddd, ^*2*^*J*=12.0, ^*3*^*J*=7.2, 5.1 Hz, 1 H), 2.97–2.83 (m, 2 H).^13^C NMR (101 MHz, CDCl_3_) δ=157.36, 136.14, 133.22, 129.38, 125.91, 124.52, 122.58, 115.87, 70.87, 54.08, 41.31, 26.03. IR υ_MAX_ (neat)/cm^−1^: 3306.34 (-O-Ar, phenol), 3062.16, 2945.47, 2854.13 (-CH_2_-, alkyl), 1655.13 (N–H stretch, amine), 1596.82, 1583.67, 1537.80, 1488.44, 1368.75, 1313.41, 1286.47, 1171.28, 1139.99, 1099.45, 1087.64, 1057.58, 1007.72, 907.50, 854.11, 826.81, 805.72, 756.78, 699.55; HRMS (ESI, *m/z*), calcd. for C_14_H_15_NO_2_SCl^+^ [M+H]^+^, 296.0512; found, 296.0519 [M+H]^+^.

#### 2.4.1.4 Ethyl (allylamino)acetate (5a)

**Figure:**
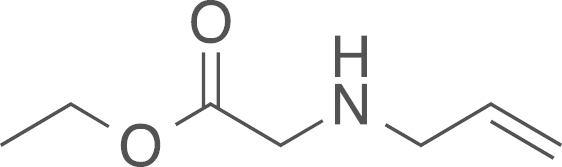

The ethyl aminoacetate (**5a**) was obtained from ethyl bromoacetate (0.66 mL, 1000 mg, 5.99 mmol, 1 eq) and allylamine (8.7 mL, 116.2 mmol, 19.4 eq) using general procedure G as a colourless oil (137 mg, 0.96 mmol, 15%). The crude material was used without further purification. ^1^H NMR (400 MHz, CDCl_3_) δ=5.86 (ddt, ^*2*^*J*=16.3, ^*3*^*J* =10.2, 6.1 Hz, 1 H), 5.21–5.08 (m, 2 H), 4.18 (q, ^*3*^*J*=7.1 Hz, 2 H), 3.39 (s, 2 H), 3.26 (dt, ^*3*^*J*=6.1, ^*4*^*J* =1.4 Hz, 2 H), 1.86 (s, N–H), 1.26 (t, ^*3*^*J*=7.1 Hz, 3 H). ^13^C NMR (101 MHz, CDCl_3_) δ=172.51, 136.01, 116.99, 60.58, 51.64, 49.81, 14.67. IR υ_MAX_ (neat)/cm^−1^: 2983.57, 2939.32 (CH_3_, -CH_2_-, alkyl), 1747.22 (C=O stretch, ester), 1647.13, 1465.64, 1421.11, 1380.02, 1216.37, 1025.87, 941.38, 854.71; HRMS (ESI, *m/z*) calcd. for C_7_H_14_NO_2_^+^ [M+H]^+^, 144.1025; found 144.1018 [M+H]^+^.

#### 2.4.1.5 Ethyl (*N*-allyl-*N*-*tert*-butoxycarbonylamino)acetate (6a)

**Figure:**
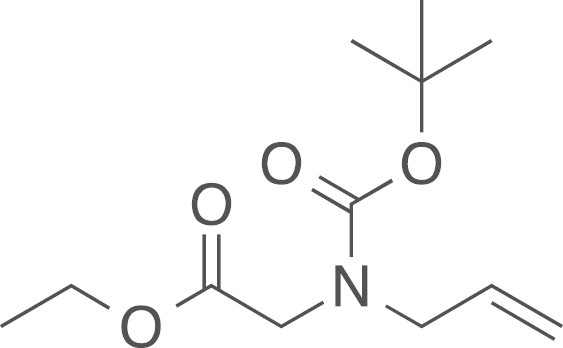

The Boc protected ethyl aminoacetate (**6a**) was obtained from ethyl (allylamino)acetate (**5a**) (137 mg, 0.96 mmol, 1 eq) and di-*tert*-butyl dicarbonate (Boc_2_O) (209 mg, 0.96 mmol, 1 eq) using general procedure H as white solid (95 mg, 0.39 mmol, 40%). The crude material was used without further purification. ^1^H NMR (400 MHz, CDCl_3_) δ=5.76 (dd, ^*2*^*J*=10.8, ^*3*^*J*=5.9 Hz, 1 H), 5.17–5.07 (m, 2 H), 4.16 (qd, ^*3*^*J*=7.1, 2.3 Hz, 2 H), 3.95–3.91 (m, 2 H), 3.86 (d, ^*2*^*J*=5.7 Hz, 1 H), 3.80 (s, 1 H), 1.43 (s, 9 H) 1.25 (q, ^*3*^*J*=6.9 Hz, 3 H); ^13^C NMR (101 MHz, CDCl_3_) δ=(C=O too weak to be detected), 133.78, 133.69, 117.60, 116.80, 80.34, 60.96, 50.79, 50.34, 48.08, 47.74, 28.30, 28.25, 14.27, 14.16; IR υ_MAX_ (neat)/cm^−1^: 2978.90, 2933.91 (CH_3_, -CH_2_-, alkyl), 1751.62 (C=O stretch, ester), 1697.69 (C=O stretch, amide), 1434.77, 1396.05, 1366.58, 1246.43, 1192.20, 1165.13, 1143.63, 1028.45, 995.74, 971.72, 927.12, 887.08, 863.60, 775.82, 716.69; HRMS (ESI, *m/z*) calcd. for C_14_H_24_N_2_O_4_Na^+^ [M+CH_3_CN+Na]^+^, 307.1634; found, 307.1650 [M+CH_3_CN+Na]^+^.

#### 2.4.1.6 (*N*-Allyl-*N*-*tert*-butoxycarbonylamino)acetic acid (7a)

**Figure:**
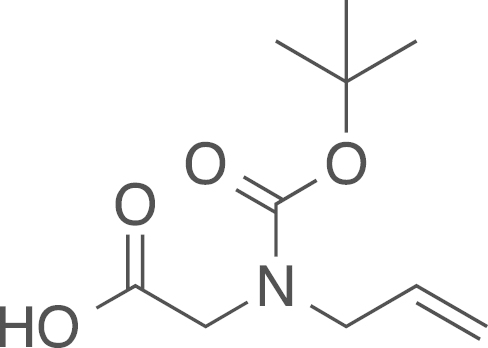

The Boc protected aminoacetic acid (**7a**) was obtained from ethyl (*N*-allyl-*N*-*tert*-butoxycarbonylamino)acetate (**6a**) (37 mg, 0.15 mmol, 1 eq) using general procedure I as colourless oil (32 mg, 0.15 mmol, 97%). ^1^H NMR (400 MHz, CDCl_3_) δ 5.78 (dt, ^*2*^*J*=10.0, ^*3*^*J*=4.5 Hz, 1 H), 5.22–5.13 (m, 2 H), 4.01–3.93 (m, 2 H), 3.91 (d, ^*2*^*J*=5.8 Hz, 2 H), 1.47 (s, 9 H);^13^C NMR (101 MHz, CDCl_3_) δ=175.15, 174.59, 155.94, 155.14, 133.50, 133.27, 117.91, 117.20, 80.92, 80.80, 50.89, 50.23, 47.62, 28.26, 28.22; IR υ_MAX_ (neat)/cm^−1^: 3077.35, 2923.95, 2796.96 (CH_3_, -CH_2_-, Alkyl), 1678.95 (C=O stretch, carboxylic acid), 1643.04 (C=O stretch, amide), 1442.16, 1419.51, 1343.26, 1260.56, 1181.01, 1095.98, 993.81, 916.01; HRMS (ESI, *m/z*) calcd. for C_10_H_17_O_4_NNa^+^ [M+Na]^+^, 238.1050; found, 238.1043 [M+Na]^+^.

#### 2.4.1.7 *N*-Allyl(2-{4-[(p-chlorophenoxy)methyl]-1-thia-5-aza-4,5,6,7-tetrahydroinden-5-yl}-2-oxoethyl)amino 2,2-dimethylpropionate(8a)

**Figure:**
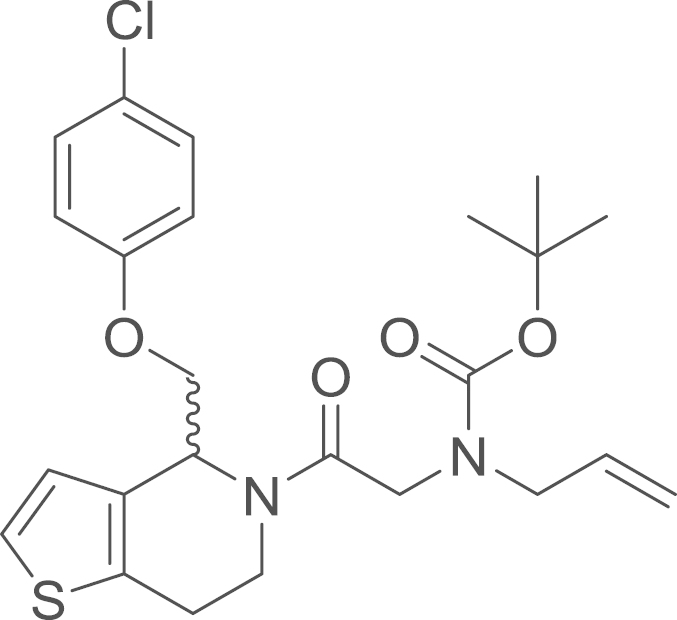

The amide (**8a**) was obtained from (4-chlorophenoxy)(1-thia-5-aza-4,5,6,7-tetrahydroinden-4-yl)methane (**4a**) (82 mg, 0.29 mmol, 1 eq) and (*N*-allyl-*N*-*tert*-butoxycarbonylamino)acetic acid (**7a**) (69 mg, 0.32 mmol, 1.1 eq) using general procedure J as yellow oil (93 mg, 0.19 mmol, 67%). ^1^H NMR (400 MHz, CDCl_3_) δ=7.28–7.16 (m, 5 H), 7.15 (d, ^*3*^*J*=4.7 Hz, 1 H), 6.94 (d, ^*3*^*J*=5.2 Hz, 1 H), 6.85 (m, 5 H), 5.83 (s, 3 H), 5.31–4.94 (m, 5 H), 4.38 (s, 1 H), 4.25 (q, ^*3*^*J*=8.4, 6.6 Hz, 3 H), 4.18–3.83 (m, 10 H), 3.62 (m, 1 H), 3.08–2.77 (m, 5 H), 1.42 (s, 18 H); ^13^C NMR (101 MHz, CDCl_3_) δ=168.55, 167.90, 157.17, 156.73, 136.62, 134.08, 132.59, 130.43, 129.44, 129.33, 125.60, 124.82, 123.81, 123.30, 116.69, 116.04, 115.92, 115.74, 80.07, 69.56, 69.09, 60.39, 53.71, 50.86, 50.38, 50.27, 50.16, 47.87, 47.77, 40.92, 35.73, 28.33, 25.65, 24.76, 21.05, 14.20.

#### 2.4.1.8 2-(Allylamino)-1-{4-[(*p*-chlorophenoxy)methyl]-1-thia-5-aza-4,5,6,7-tetrahydroinden-5-yl}-1-ethanone (9a)

**Figure:**
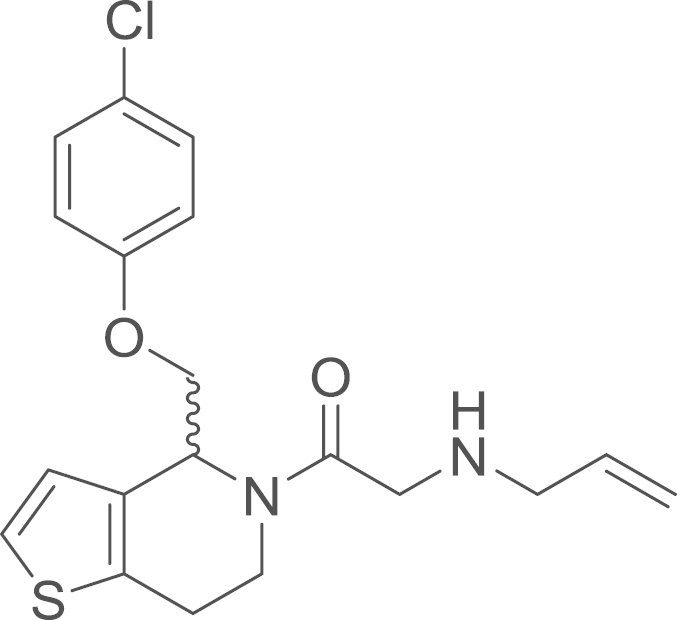

RU-SKI 41 (**9a**) was obtained from *N*-allyl(2-{4-[(*p*-chlorophenoxy)methyl]−1-thia−5-aza−4,5,6,7-tetrahydroinden−5-yl}−2-oxoethyl)amino 2,2-dimethylpropionate (**8a**) (86 mg, 0.18 mmol, 1 eq) using general procedure M as yellow oil (50 mg, 0.13 mmol, 73%). ^1^H NMR (400 MHz,CDCl_3_, rotameric ratio *E*:*Z* 1:1) δ 7.28–7.21 (m, 5 H), 7.18 (d, ^*3*^*J*=5.2 Hz, 1 H), 6.96 (d, ^*3*^*J*=5.2 Hz, 1 H), 6.92 (d, ^*3*^*J*=5.2 Hz, 1 H), 6.87–6.81 (m, 4 H), 5.99–5.86 (m, 3 H), 5.27–5.20 (m, 3 H), 5.14 (d, ^*3*^*J*=10.3 Hz, 2 H), 5.06–5.00 (m, 1 H), 4.25 (dt, ^*3*^*J*=7.2, 3.4 Hz, 2 H), 4.19–4.11 (m, 2 H), 4.05–3.98 (m, 1 H), 3.85 (d, ^*2*^*J*=16.1 Hz, 1 H), 3.72–3.52 (m, 6 H), 3.51 (s, 3 H), 3.32 (m, 4 H), 3.04 (qd, ^*2*^*J*=12.1, ^*3*^*J*=4.4 Hz, 2 H), 2.95 (m, 3 H), 2.89–2.81 (m, 2 H); ^13^C NMR (101 MHz, CDCl_3_) δ=171.08, 170.22, 157.14, 156.67, 136.64, 136.54, 136.39, 133.82, 132.57, 130.48, 129.52, 129.34, 126.45, 125.96, 125.56, 124.88, 123.90, 123.40, 116.52, 116.34, 115.91, 115.68, 69.59, 69.15, 53.64, 52.25, 52.20, 50.76, 50.05, 49.84, 40.61, 35.86, 25.64, 24.80; ^13^C NMR (DEPT) (101 MHz, CDCl_3_) δ=136.53, 136.39, 129.52, 129.34, 125.56, 124.88, 123.91, 123.41, 116.53, 116.35, 115.90, 115.67, 69.59, 69.14, 53.64, 52.25, 52.20, 50.76, 50.05, 49.83, 40.61, 35.86, 25.64, 24.80; IR υ_MAX_ (neat)/cm^−1^: 2905.66 (CH_3_, -CH_2_-, Alkyl), 1644.44 (C=O stretch, amide), 1491.88, 1427.41, 1284.32, 1242.08, 1212.27, 1170.89, 1092.26, 1046.83, 1006.02, 921.03, 825.66, 744.68, 710.72, 660.29; HRMS (ESI, *m/z*) calcd. for C_19_H_21_ClN_2_O_2_S^+^ [M+H]^+^, 377.1091; found, 377.1109 [M+H]^+^.

### 2.4.2 RU-SKI 43 synthetic data

#### 2.4.2.1 Ethyl (*m*-tolyloxy)acetate (1b)

**Figure:**
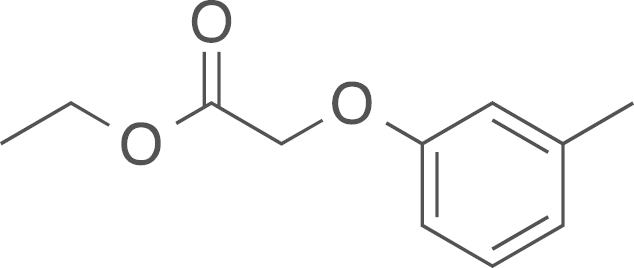

The ethyl (*m*-tolyloxy)acetate (**1b**) was obtained from *m*-cresol (0.63 mL, 0.65 mg, 5.99 mmol, 1 eq) and 2-bromoethylacetate (0.66 mL, 1000 mg, 5.99 mmol, 1 eq) using general procedure A as a white solid (1.09 g, 5.61 mmol, 93%). ^1^H NMR (400 MHz, CDCl_3_) δ=7.17 (t, ^*3*^*J*=7.9 Hz, 1 H), 6.81 (d, ^*3*^*J*=7.5 Hz, 1 H), 6.74 (s, 1 H), 6.70 (dd, ^*2*^*J*=8.2, ^*3*^*J*=2.4 Hz, 1 H), 4.60 (s, 2 H), 4.27 (q, ^*3*^*J*=7.1 Hz, 2 H), 2.32 (s, 3 H), 1.30 (t, ^*3*^*J*=7.2 Hz, 3 H), ^13^C NMR (101 MHz, CDCl_3_) δ=169.52, 158.28, 140.11, 129.71, 123.02, 116.05, 111.85, 65.85, 61.76, 21.94, 21.50; IR υ_MAX_ (neat)/cm^−1^: 2984.65, 2924.90 (CH_3_, -CH_2_-, Alkyl), 2924.90, 1758.38, 1734.58 (C=O, ester), 1586.99, 1489.76, 1442.80, 1378.32, 1275.60, 1261.07, 1200.37, 1156.26, 1088.71, 1029.60, 912.07, 857.19, 765.50, 750.32, 689.03; HRMS (ESI, *m/z*) calcd. for C_1_^1^H_15_O_3_^+^ [M+H]^+^, 195.1021; found, 195.1023 [M+H]^+^.

#### 2.4.2.2 1-[2-(2-Thienyl)ethylamino]-2-(*m*-tolyloxy)-1-ethanone (2b)

**Figure:**
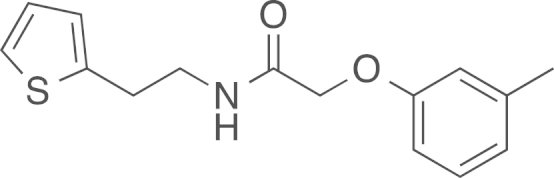

The amide (**2b**) was obtained from 2-(3-thienyl)ethylamine (0.48 mL, 522 mg, 4.08 mmol, 1 eq) and ethyl (*m*-tolyloxy)acetate (793 mg, 4.08 mmol, 1 eq) using general procedure B as a white solid (853 mg, 3.10 mmol, 76%). ^1^H NMR (400 MHz, CDCl_3_) δ=7.19 (d, ^*3*^*J*=7.8 Hz, 1 H), 7.16 (ddd, ^*3*^*J*=5.0, 3.0, ^*4*^*J*=1.0 Hz, 1 H), 6.94 (ddd, ^*3*^*J*=7.5, 5.1, ^*4*^*J*=3.4 Hz, 1 H), 6.86–6.82 (m, 1 H), 6.79–6.76 (m, 1 H), 6.71–6.65 (m, 2 H), 4.47 (s, 2 H), 3.62 (q, ^*3*^*J*=6.6 Hz, 2 H), 3.07 (t, ^*3*^*J*=6.7 Hz, 2 H), 2.34 (s, 3 H); ^13^C NMR (101 MHz, CDCl_3_) δ=168.36, 157.19, 142.21, 140.81, 129.51, 127.06, 125.49, 124.07, 122.93, 115.44, 111.53, 67.30, 43.63, 40.28, 29.91; IR υ_MAX_ (neat)/cm^−1^: 3103.09 (-O-Ar, br, phenol), 3046.00, 2922.20, 2860.65 (CH_3_, -CH_2_-, alkyl), 1663.53 (C=O stretch, amide), 1586.94, 1532.30, 1488.90, 1437.13, 1365.63, 1258.27, 1157.44, 1067.43, 920.48, 849.56, 824.35, 765.74, 750.20, 689.79; HRMS (ESI, *m/z*) calcd. for C_15_H_18_NO_2_S^+^ [M+H]^+^, 276.1058; found, 276.1071 [M+H]^+^.

#### 2.4.2.3 (1-Thia-5-aza-4,5,6,7-tetrahydroinden-4-yl)(*m*-tolyloxy)methane (4b) (ring closured imine 3b)/(amine 4b)

**Figure:**
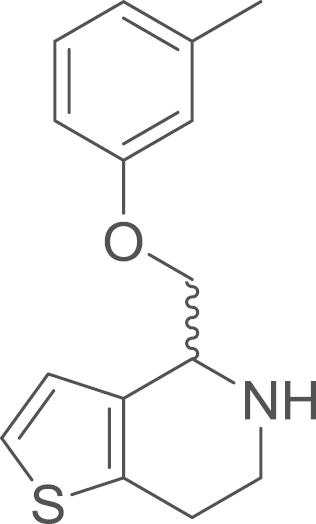

The cyclic imine (1-thia-5-aza-6,7-dihydroinden-4-yl)(*m*-tolyloxy)methane (**3b**) was obtained from 1-[2-(2-thienyl)ethylamino]-2-(*m*-tolyloxy)-1-ethanone (**2b**) (807 mg, 2.93 mmol, 1 eq) using general procedure E as an orange oil (476 mg, 1.85 mmol, 63%). The crude material was used without further purification. ^1^H NMR (400 MHz, CDCl_3_) δ=7.33–7.25 (m, 1 H), 7.23–7.05 (m, 2 H), 7.03–6.93 (m, 1 H), 6.83 (s, 1 H), 6.83–6.75 (m, 1 H), 4.98 (s, 2 H), 3.91 (ddt, ^*2*^*J*=9.7, ^*3*^*J*=7.3, 1.2 Hz, 2 H), 2.92 (m, 2 H), 2.30 (s, 3 H). The cyclic amine (**4b**) was obtained from (1-thia-5-aza-6,7-dihydroinden-4-yl)(*m*-tolyloxy)methane (**3b**) (476 mg, 1.85 mmol, 1 eq) using general procedure F as a yellow solid (235 mg, 0.91 mmol, 49%). ^1^H NMR (400 MHz, CDCl_3_) δ=7.20 (t, ^*3*^*J*=7.9 Hz, 1 H), 7.14 (d, ^*3*^*J*=5.2 Hz, 1 H), 6.92 (d, ^*3*^*J*=5.2 Hz, 1 H), 6.81 (t, ^*3*^*J*=6.5 Hz, 3 H), 4.43–4.38 (m, 1 H), 4.24 (dd, ^*2*^*J*=9.2, ^*3*^*J*=3.8 Hz, 1 H), 4.11–4.06 (m, 1 H), 3.34 (dt, ^*2*^*J*=12.0, ^*3*^*J*=5.2 Hz, 1 H), 3.12 (ddd, ^*2*^*J*=12.1, ^*3*^*J*=6.9, 5.2 Hz, 1 H), 2.90 (tt, ^*2*^*J*=10.7, ^*3*^*J*=5.2 Hz, 2 H), 2.36 (s, 3 H), 2.31 (s, 1 H, N–H); ^13^C NMR (101 MHz, CDCl_3_) δ=158.74, 139.55, 136.00, 133.55, 129.23, 124.67, 122.42, 121.84, 115.44, 111.48, 70.30, 54.20, 41.23, 26.07, 21.54; IR υ_MAX_ (neat)/cm^−1^ 2919.94, 2865.60, 2837.79 (alkyl, CH_3_, -CH_2_-), 1600.89 (N–H stretch, amine), 1584.04, 1488.99, 1466.59, 1433.15, 1382.23, 1359.82, 1326.95, 1311.57, 1290.14, 1259.12, 1170.61, 1156.70, 1132.10, 1047.23, 1032.89, 994.75, 879.52, 827.82, 769.24, 752.00, 711.74, 688.35; HRMS (ESI, *m/z*) calcd. for C_15_H_18_NOS^+^ [M+H]^+^, 260.1109; found, 260.1112 [M+H]^+^.

#### 2.4.2.4 Ethyl (2-methylbutylamino)acetate (5b)

**Figure:**
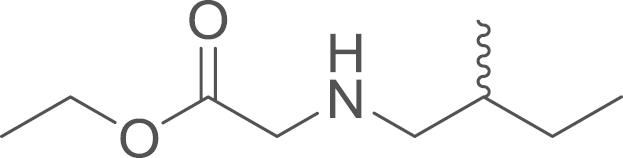

The ethyl aminoacetate (**5b**) was obtained from ethyl bromoacetate (166 μL 250 mg, 1.50 mmol, 1 eq) and 2-methyl butylamine (3.3 mL, 2.53 g, 29.04 mmol, 1 eq) using general procedure G. The crude material was purified by Isolera (SiO_2_; EtOAc:Hexane:TEA-12:88:1) and the expected compound was recovered as colourless oil (152 mg, 0.88 mmol, 58%).^1^H NMR (400 MHz, CDCl_3_) δ=4.16 (q, ^*3*^*J*=7.1 Hz, 2 H), 3.35 (s, 2 H), 2.59 (dd, ^*2*^*J*=12.6, ^*3*^*J*=5.5 Hz, 1 H), 2.52–2.46 (dd, ^*2*^*J*=11.2, ^*3*^*J*=7.1 Hz 1 H), 2.35 (dd, ^*2*^*J*=11.2, ^*3*^*J*=7.1 Hz, 1 H), 1.53–1.32 (m, 2 H), 1.25 (t, ^*3*^*J*=7.1 Hz, 3 H), 0.91–0.82 (m, 6 H); ^13^C NMR (101 MHz, CDCl_3_) δ=172.62, 60.65, 55.70, 51.24, 34.95, 27.32, 17.56, 11.23; HRMS (ESI, *m/z*) calcd. for C_9_H_20_NO_2_^+^ [M+H]^+^, 174.1494; found, 174.1497 [M+H]^+^.

#### 2.4.2.5 Ethyl [*N*-*tert*-butoxycarbonyl(2-methylbutyl)amino]acetate (6b)

**Figure:**
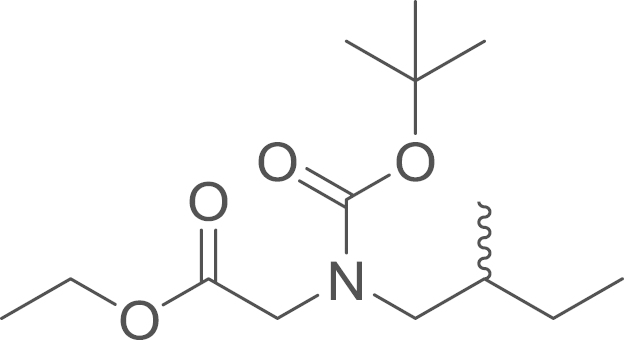

The Boc protected ethyl aminoacetate (**6b**) was obtained from ethyl (2-methylbutylamino)acetate (**5b**) (152 mg, 0.88 mmol, 1 eq) and di-*tert*-butyl dicarbonate (Boc_2_O) (427 mg, 1.955 mmol, 2.2 eq) using general procedure H. The crude material was purified by Isolera (SiO2; EtOAc:Hexane-2:8) and the expected compound was obtained as white solid (139 mg, 0.51 mmol, 57%). ^1^H NMR (400 MHz, CDCl_3_) δ 4.21 (qd, ^*3*^*J*=7.1, 3.1 Hz, 2 H), 4.02–3.90 (m, 1 H), 3.86 (s, 1 H), 3.20 (dd, ^*2*^*J*=14.2, ^*3*^*J*=6.7 Hz, 1 H), 3.08 (ddd, ^*2*^*J*=32.8, ^*3*^*J*=14.2, 8.0 Hz, 1 H), 1.62 (dd, ^*2*^*J*=13.7, ^*3*^*J*=5.7 Hz, 1 H), 1.47 (s, 9 H), 1.41 (d, ^*3*^*J*=6.3 Hz, 1 H), 1.30 (q, ^*3*^*J*=7.2 Hz, 3 H), 1.13 (dt, ^*2*^*J*=13.8, ^*3*^*J*=7.3 Hz, 1 H), 0.95–0.87 (m, 6 H); ^13^C NMR (101 MHz, CDCl_3_) δ=170.25, 170.11, 156.15, 155.57, 80.01, 79.92, 60.93, 54.39, 54.35, 49.99, 49.32, 34.26, 33.97, 28.35, 28.25, 27.41, 26.89, 17.03, 16.81, 14.26, 14.15, 11.31; IR υ_MAX_ (neat)/cm^−1^: 2966.24, 2932.72, 2877.14 (CH_3_, -CH_2_-, alkyl), 1753.57 (C=O stretch, ester), 1695.10 (C=O stretch, amide), 1456.93, 1403.46, 1365.89, 1245.03, 1192.65, 1174.47, 1145.09, 1096.17, 1029.26, 969.04, 930.01, 886.08, 863.78, 775.68; HRMS (ESI, *m/z*): calcd. for C_16_H_30_N_2_O_4_Na^+^ [M+CH_3_CN+Na]^+^, 337.2103; found, 337.2112 [M+CH_3_CN+Na]^+^.

#### 2.4.2.6 [*N*-*tert*-Butoxycarbonyl(2-methylbutyl)amino]acetic acid (7b)

**Figure:**
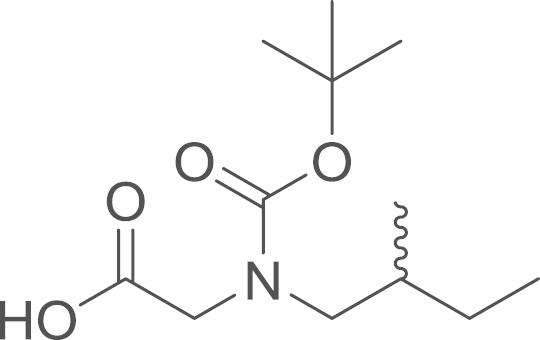

The Boc protected aminoacetic acid (**7b**) was obtained from ethyl [*N*-*tert*-butoxycarbonyl(2-methylbutyl)amino]acetate (**6b**) (51 mg, 0.19 mmol, 1 eq) using general procedure I as colourless oil (44 mg, 0.18 mmol, 95%). ^1^H NMR (400 MHz, CDCl_3_) δ=4.00 (d, ^*3*^*J*=4.0 Hz, 1 H), 3.92 (s, 1 H), 3.21 (dd, ^*2*^*J*=14.3, ^*3*^*J*=6.6 Hz, 1 H), 3.16–3.02 (m, 1 H), 1.64 (s, 1 H), 1.47 (s, 9 H), 1.42–1.25 (m, 1 H), 1.13 (dt, ^*2*^*J*=14.5, ^*3*^*J*=7.6 Hz, 1 H), 0.91 (m, 6 H); ^13^C NMR (101 MHz, CDCl_3_) δ=175.72, 174.65, 156.70, 155.49, 80.80, 80.38, 77.34, 77.23, 77.02, 76.88, 76.71, 54.62, 54.23, 49.50, 34.19, 33.94, 28.32, 28.23, 26.86, 16.96, 16.80, 11.29; IR υ_MAX_ (neat)/cm^−1^: 2965.36, 2933.32, 2877.19 (Alkyl, CH_3_, -CH_2_-), 1695.08 (br, C=O stretch merged together, carboxylic acid and carbamate), 1461.49, 1423.92, 1403.69, 1366.79, 1246.53, 1147.12, 1098.08, 969.73, 927.38, 871.21, 766.79; HRMS (ESI, *m/z*) calcd. for C_14_H_26_N_2_O_4_Na^+^ [M+CH_3_CN+Na]^+^, 309.1785; found, 309.1806 [M+CH_3_CN+Na]^+^.

#### 2.4.2.7 (2-Oxo−2-{4-[(*m*-tolyloxy)methyl]−1-thia−5-aza−4,5,6,7-tetrahydroinden−5-yl}ethyl)(2-methylbutyl)amino 2,2-dimethylpropionate (8b)

**Figure:**
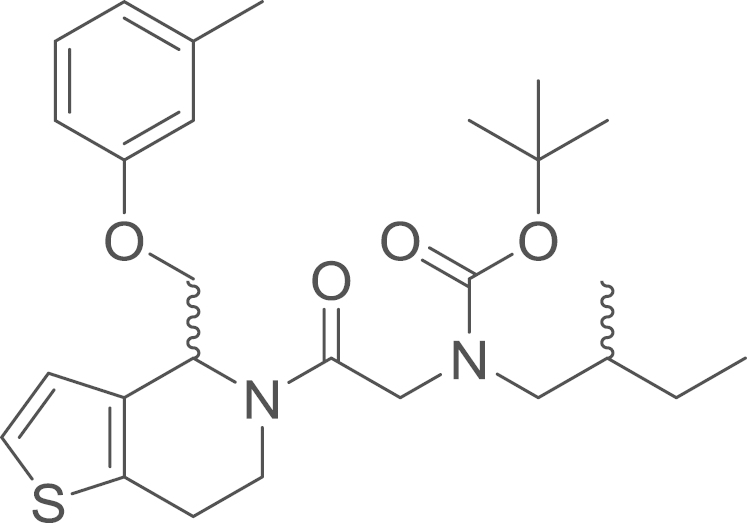

The amide (**8b**) was obtained from (1-thia−5-aza−4,5,6,7-tetrahydroinden−4-yl)(m-tolyloxy)methane (**4b**) (31 mg, 0.12 mmol, 1 eq) and (**7b**) [*N*-*tert*-butoxycarbonyl(2-methylbutyl)amino]acetic acid (32.3 mg, 0.13 mmol, 1.1 eq) using general procedure J as yellow oil (21 mg, 0.045 mmol, 37%). ^1^H NMR (400 MHz, CDCl_3_) δ 7.23–7.13 (m, 4 H), 6.97 (d, ^*3*^*J*=5.3 Hz, 1 H), 6.91 (dd, ^*3*^*J*=15.3, 5.2 Hz, 1 H), 6.82 (t, ^*3*^*J*=6.2 Hz, 1 H), 6.77 (d, ^*3*^*J*=6.8 Hz, 2 H), 6.70 (d, ^*3*^*J*=8.0 Hz, 3 H), 5.31–5.22 (m, 1 H), 4.99 (dt, ^*2*^*J*=11.6, ^*3*^*J*=5.5 Hz, 1 H), 4.57–4.43 (m, 1 H), 4.37–4.23 (m, 3 H), 4.14 (m, 3 H), 3.69 (dd, ^*2*^*J*=17.9, ^*3*^*J*=7.2 Hz, 1 H), 3.35 (dtd, ^*2*^*J*=42.4, ^*3*^*J*=14.3, 7.6 Hz, 3 H), 3.23–2.76 (m, 6 H), 2.33 (s, 6 H), 1.54–1.25 (m, 25 H), 1.20–1.05 (m, 2 H), 0.92 (dp, ^*3*^*J*=9.8, 3.1 Hz, 12 H); ^13^C NMR (101 MHz, CDCl_3_) δ=171.14, 167.83, 158.59, 158.09, 156.42, 139.71, 139.45, 136.59, 133.76, 132.90, 130.68, 129.32, 129.15, 125.68, 124.90, 123.69, 123.10, 122.25, 121.77, 115.52, 115.38, 111.26, 111.04, 110.95, 79.99, 79.67, 69.31, 69.21, 68.93, 68.66, 54.29, 54.13, 53.73, 51.10, 49.27, 48.83, 40.97, 35.62, 34.05, 33.83, 28.40, 28.26, 27.00, 25.69, 24.80, 21.46, 17.12, 16.90, 11.45, 11.34; IR υ_MAX_ (neat)/cm^−1^: 2962.99, 2926.50, 2874.48 (CH_3_, -CH_2_-, alkyl), 1694.23 (C=O stretch, carbamate), 1661.69 (C=O stretch, amide), 1602.85, 1585.14, 1460.59, 1433.04, 1364.90, 1260.21, 1246.48, 1208.04, 1156.84, 1095.12, 1053.60, 968.15, 927.76, 878.13, 839.75, 765.50, 690.83; HRMS (ESI, *m/z*) calcd. for C_27_H_39_N_2_O_4_S^+^ [M+H]^+^, 487.2625; found, 487.2621 [M+H]^+^.

#### 2.4.2.8 2-(2-Methylbutylamino)-1-{4-[(*m*-tolyloxy)methyl]-1-thia-5-aza-4,5,6,7-tetrahydroinden-5-yl}-1-ethanone (9b)

**Figure:**
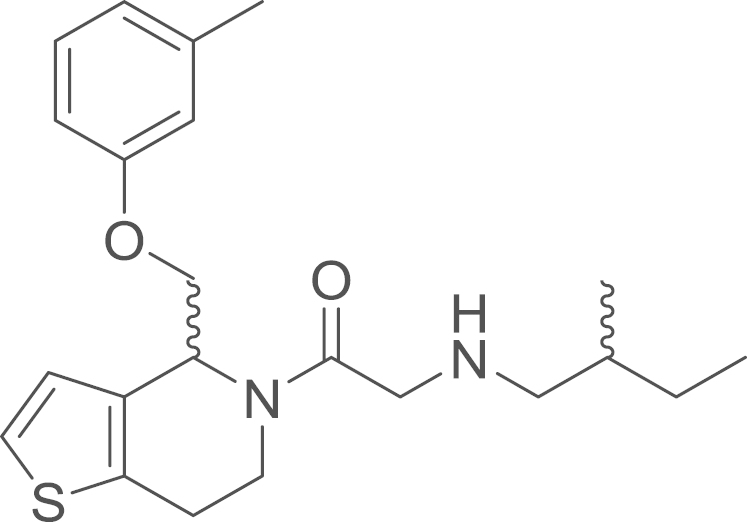

RU-SKI 43 (**9b**) was obtained from *N*-allyl(2-{4-[(*p*-chlorophenoxy)methyl]-1-thia-5-aza-4,5,6,7-tetrahydroinden-5-yl}-2-oxoethyl)amino 2,2-dimethylpropionate (**8b**) (15.8 mg, 0.033 mmol, 1 eq) using general procedure L as yellow oil (8.1 mg, 0.021 mmol, 62%). ^1^H NMR (400 MHz, CDCl_3_, rotameric ratio *E*:*Z* 4:6) δ=7.24–7.13 (m, 4 H), 6.98 (d, ^*3*^*J*=5.2 Hz, 1 H), 6.93 (d, ^*3*^*J*=5.2 Hz, 1 H), 6.82 (d, ^*3*^*J*=7.5 Hz, 1 H), 6.77 (d, ^*3*^*J*=7.6 Hz, 1 H), 6.73–6.67 (m, 4 H), 5.89 (t, ^*3*^*J*=4.7 Hz, 1 H), 5.27 (dd, ^*2*^*J*=8.8, ^*3*^*J*=4.1 Hz, 1 H), 5.01 (dd, ^*2*^*J*=12.8, ^*3*^*J*=4.5 Hz, 1 H), 4.31–4.24 (m, 2 H), 4.22–4.12 (m, 2 H), 4.06–4.00 (m, 1 H), 3.89 (dd, ^*2*^*J*=16.0, ^*3*^*J*=2.0 Hz, 1 H), 3.73–3.49 (m, 4 H), 3.07 (td, ^*2*^*J*=12.2, ^*3*^*J*=3.9 Hz, 1 H), 2.96 (dq, ^*2*^*J*=16.8, ^*3*^*J*=6.5, 5.7 Hz, 2 H), 2.87–2.81 (m, 1 H), 2.58 (tdd, ^*3*^*J*=9.7, 6.1, ^*4*^*J*=3.8 Hz, 2 H), 2.51–2.41 (m, 2 H), 2.34 (s, 3 H), 2.32 (s, 3 H), 1.54 (dddd, ^*2*^*J*=40.6, ^*3*^*J*=19.2, 12.0, 5.2 Hz, 4 H), 1.19 (dtd, ^*2*^*J*=14.8, ^*3*^*J*=7.4, 4.0 Hz, 2 H), 1.00–0.89 (m, 11 H); ^13^C NMR (101 MHz, CDCl_3_) δ=170.97, 170.18, 158.55, 158.05, 139.75, 139.47, 136.47, 133.69, 132.85, 130.76, 129.34, 129.15, 125.64, 124.97, 123.77, 123.19, 122.29, 121.81, 115.50, 115.28, 111.28, 111.04, 69.22, 68.66, 56.31, 53.73, 51.52, 50.96, 50.91, 40.68, 35.81, 35.02, 34.91, 27.43, 27.35, 25.65, 24.81, 21.46, 17.65, 17.57, 11.29, 11.21; HRMS (ESI, *m/z*) calcd. for C_22_H_31_N_2_O_2_S^+^ [M+H]^+^, 387.2101; found, 387.2109 [M+H]^+^.

### 2.4.3 RU-SKI 101 Synthetic Data

#### 2.4.3.1 4-(*m*-Tolyl)-1-thia-5-aza-4,5,6,7-tetrahydroindene (4c)

**Figure:**
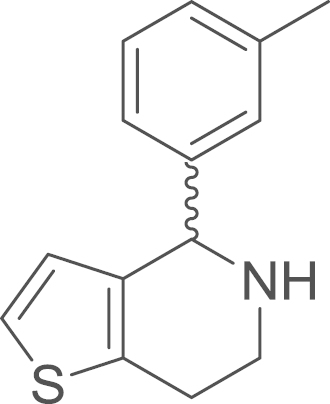

The amine was synthesised as previously described [4].

#### 2.4.3.2 2-(2-Methylbutylamino)-1-[4-(*m*-tolyl)-1-thia-5-aza-4,5,6,7-tetrahydroinden-5-yl]-1-ethanone (8c)

**Figure:**
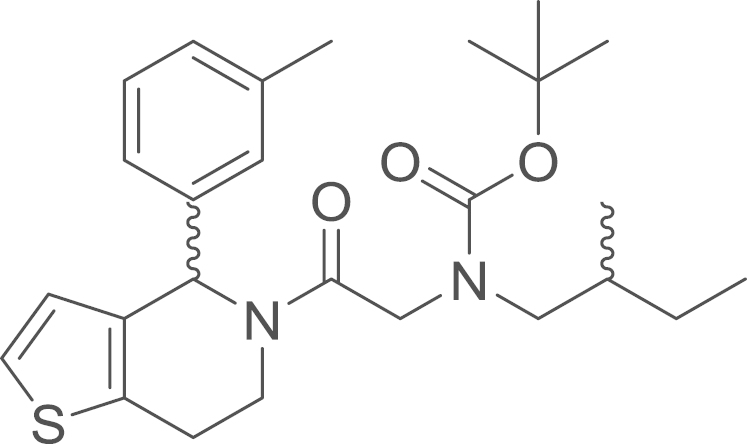

The amide (**8c**) was obtained from 4-(*m*-tolyl)-1-thia-5-aza-4,5,6,7-tetrahydroindene (**4c**) (28 mg, 0.12 mmol, 1 eq) and [*N*-*tert*-butoxycarbonyl(2-methylbutyl)amino]acetic acid (**7b**) (30 mg, 0.12 mmol, 1 eq) using general procedure K as a yellow oil (35 mg, 0.08 mmol, 64%). Rf 0.14 (SiO_2_; EtOAc:Hex, 1:9). ^1^H NMR (400 MHz, CDCl3) δ 7.17 (m, 3 H), 7.11 (d, ^*3*^*J*=7.4 Hz, 1 H), 7.05 (d, ^*3*^*J*=7.6 Hz, 1 H), 6.84 (s, 1 H), 6.71 (s, 1 H), 4.33 (t, ^*3*^*J*=14.2 Hz, 1 H), 4.10–3.72 (m, 2 H), 3.39 (s, 1 H), 3.28–2.79 (m, 4 H), 2.33 (s, 3 H), 1.68 (s, 1 H), 1.49 (s, 6 H), 1.35 (s, 3 H), 1.28 (s, 1 H), 1.10 (s, 1 H), 0.96–0.74 (m, 6 H).13 C NMR, HRMS (ESI, m/z)

#### 2.4.3.3 2-(2-Methylbutylamino)-1-[4-(*m*-tolyl)-1-thia-5-aza-4,5,6,7-tetrahydroinden-5-yl]-1-ethanone (9c)

**Figure:**
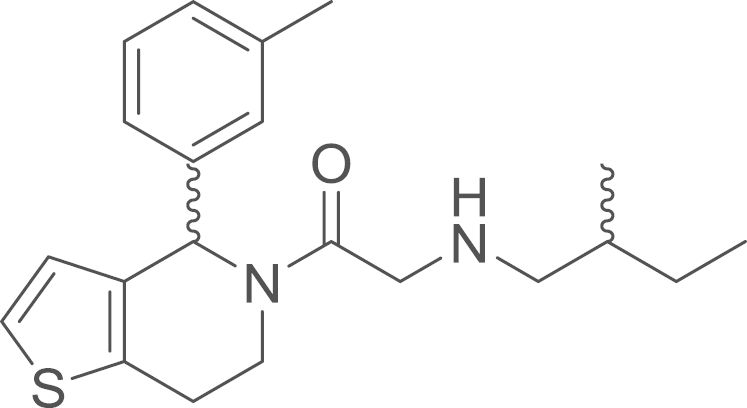

RU-SKI 101 (**9c**) was obtained from 2-(2-methylbutylamino)-1-[4-(*m*-tolyl)-1-thia-5-aza-4,5,6,7-tetrahydroinden-5-yl]-1-ethanone (**8c**) (44 mg, 0.10 mmol, 1 eq) using general procedure L as yellow oil (18 mg, 0.05 mmol, 52%). ^1^H NMR (400 MHz, CDCl_3_, rotameric ratio *E*:*Z* 2 :8) δ= 7.27–6.98 (m, 7 H), 6.87 (s, 1 H), 6.77 (d, ^*3*^*J*=5.3 Hz, 1 H), 6.72 (d, ^*3*^*J*=5.2 Hz, 1 H), 5.90 (s, 1 H), 4.89 (d, ^*3*^*J*=8.9 Hz, 1 H), 3.81 (dd, ^*2*^*J*=14.1, ^*3*^*J*=5.0 Hz, 1 H), 3.53 (qd, ^*2*^*J*=15.8, ^*3*^*J*=4.4 Hz, 2 H), 3.37 (td, ^*2*^*J*=14.1, ^*3*^*J*=4.3 Hz, 1 H), 3.04 (ddd, ^*2*^*J*=16.8, ^*3*^*J*=11.4, 5.6 Hz, 2 H), 2.97–2.83 (m, 2 H), 2.58 (dt, ^*2*^*J*=11.2, ^*3*^*J*=5.6 Hz, 1 H), 2.45 (ddd, ^*3*^*J*=10.9, 7.2, ^*4*^*J*=2.5 Hz, 2 H), 2.33 (s, 4 H), 2.27–2.20 (m, N–H), 1.59 (dp, ^*2*^*J*=13.3, ^*3*^*J*=6.6 Hz, 1 H), 1.47 (dq, ^*2*^*J*=13.0, ^*3*^*J*=6.5, 5.6 Hz, 2 H), 1.19 (dt, ^*2*^*J*=14.6, ^*3*^*J*=6.5 Hz, 2 H), 0.93 (dt, ^*2*^*J*=14.8, ^*3*^*J*=7.0 Hz, 9 H); ^13^C NMR (101 MHz, CDCl_3_) δ=169.20, 144.73, 140.82, 138.15, 134.12, 134.00, 133.67, 129.35, 128.82, 128.62, 128.18, 126.63, 126.07, 125.77, 125.68, 124.50, 123.36, 123.28, 56.32, 54.02, 51.21, 38.51, 34.99, 34.96, 27.46, 27.36, 25.74, 21.48, 17.65, 11.34, 11.29; IR υ_MAX_ (neat)/cm^−1^: 2958.22, 2922.15, 2874.72 (CH3, -CH_2_-, alkyl), 1646.21 (C=O, amide), 1461.28, 1424.10, 1379.20, 1332.27, 1289.10, 1208.03, 1172.73, 1152.07, 1044.79, 892.05, 837.88, 810.68, 767.27, 736.91, 708.37; HRMS (ESI, *m/z*) calcd. for C_2_^1^H_29_N_2_OS^+^ [M+H]^+^, 357.1995; found, 357.2019 [M+H]^+^.

## 2.5 RU-SKI 201 synthetic data

### 2.5.1 (6-Methyl-2-pyridyl)[2-(2-thienyl)ethylamino]formaldehyde (2d)

**Figure:**
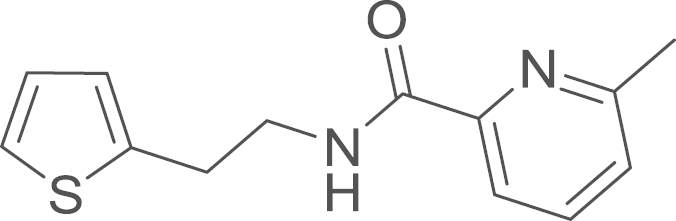

The amide (**2d**) was obtained from 2-(3-thienyl)ethylamine (390 uL, 424 mg, 3.31 mmol, 1 eq) and 6-methylpyridine-2-carboxylic acid (500 mg, 3.65 mmol, 1.1 eq) using general procedure C as a colourless oil (650 mg, 2.64 mmol, 72%). ^1^H NMR (400 MHz, CDCl_3_) δ=8.35 (s, 1 H, N–H), 8.02 (d, ^*3*^*J*=7.7 Hz, 1 H), 7.73 (t, ^*3*^*J*=7.7 Hz, 1 H), 7.28 (d, ^*3*^*J*=7.4 Hz, 1 H), 7.19 (d, ^*3*^*J*=5.1 Hz, 1 H), 7.00–6.96 (m, 1 H), 6.92 (d, ^*3*^*J*=3.2 Hz, 1 H), 3.77 (q, ^*3*^*J*=6.8 Hz, 2 H), 3.19 (t, ^*3*^*J*=7.0 Hz, 2 H), 2.56 (s, 3 H); ^13^C NMR (101 MHz, CDCl_3_) δ=164.55, 157.12, 149.11, 141.35, 137.46, 127.01, 125.87, 125.33, 123.85, 119.21, 40.86, 30.14, 24.25; IR υ_MAX_ (neat)/cm^−1^: 3256.7 (C–H stretch, Ar–H), 2920.3, 2902.4, 2824, 2806 (CH_3_, -CH_2_-, alkyl), 1667.66 (C=O stretch, amide), 1593.76, 1518.38, 1490.74, 1451.76, 1330.58, 1278.30, 1210.09, 1198.23, 1112.15, 1082.07, 1071.68.1052.13, 1025.33, 984.79, 931.22, 915.08, 901.76, 867.48, 853.24, 840.63, 823.83, 759.51, 726.94, 700.82, 686.66; HRMS (ESI, *m/z*) calcd. for C_13_H_15_N_2_OS^+^ [M+H]^+^, 247.0905; found, 247.0916 [M+H]^+^.

### 2.5.2 4-(6-Methyl-2-pyridyl)-1-thia-5-aza-4,5,6,7-tetrahydroindene (4d) (ring closured imine 3d)/(amine 4d)

**Figure:**
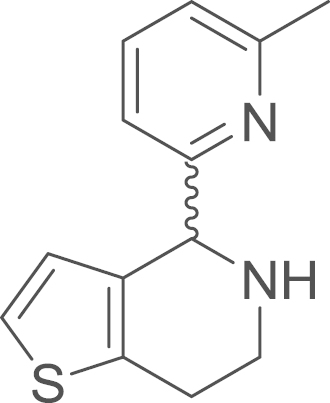

The cyclic imine (**3d**) was obtained from (6-methyl-2-pyridyl)[2-(2-thienyl)ethylamino]formaldehyde (**2d**) (610 mg, 4.76 mmol, 1 eq) using general procedure E as a brown oil (478 mg, 2.10 mmol, 84%). The crude material was used without further purification. ^1^H NMR (400 MHz, CDCl_3_) δ=8.03 (d, ^*3*^*J*=7.6 Hz, 1 H), 7.82 (d, ^*3*^*J*=7.7 Hz, 1 H), 7.58 (d, ^*3*^*J*=5.2 Hz, 1 H), 7.02 (d, ^*3*^*J*=5.9 Hz, 1 H), 6.99 (dd, ^*3*^*J*=5.1, 3.4 Hz, 1 H), 4.08–4.03 (m, 2 H), 3.03–2.96 (m, 2 H), 2.29 (s, 3 H); The amine (**4d**) was obtained from 4-(6-methyl-2-pyridyl)-1-thia-5-aza-6,7-dihydroindene (**3d**) (468 mg, 2.05 mmol, 1 eq) using general procedure G as brown oil (281 mg, 1.22 mmol, 49% over two steps). ^1^H NMR (400 MHz, CDCl_3_) δ=7.52 (t, ^*3*^*J*=7.7 Hz, 1 H), 7.05 (dd, ^*3*^*J*=6.4, 3.2 Hz, 3 H), 6.98 (d, ^*3*^*J*=7.7 Hz, 1 H), 6.62 (d, ^*3*^*J*=5.2 Hz, 1 H), 5.15 (s, 1 H), 3.28 (dt, ^*2*^*J*=12.2, ^*3*^*J*=5.1 Hz, 1 H), 3.14 (ddd, ^*2*^*J*=12.3, ^*3*^*J*=7.6, 5.1 Hz, 1 H), 3.02–2.86 (m, 2 H), 2.57 (s, 3 H); ^13^C NMR (101 MHz, CDCl_3_) δ=161.30, 158.08, 136.70, 135.27, 135.18, 126.13, 122.00, 121.95, 119.29, 60.83, 41.90, 25.88, 24.51; IR υ_MAX_ (neat)/cm^−1^: 3289.25, 3044.04 (C–H stretch, Ar–H), 2923.00 (CH_3_, -CH_2_-, alkyl), 1592.05 (N–H stretch, amine), 1574.69 (N–H stretch, amine), 1442.07, 1372.97, 1260.56, 1208.46, 1158.68, 1146.21, 1123.21, 1090.07, 1032.98, 991.98, 934.93, 873.05, 851.80, 797.99, 766.03, 739.69, 712.11; HRMS (ESI, *m/z*) calcd. for C_13_H_15_N_2_S^+^ [M+H]^+^, 231.0950; found, 231.0959 [M+H]^+^.

### 2.5.3 {2-[4-(6-Methyl-2-pyridyl)-1-thia-5-aza-4,5,6,7-tetrahydroinden-5-yl]-2-oxoethyl}(2-methylbutyl)amino 2,2-dimethylpropionate (8d)

**Figure:**
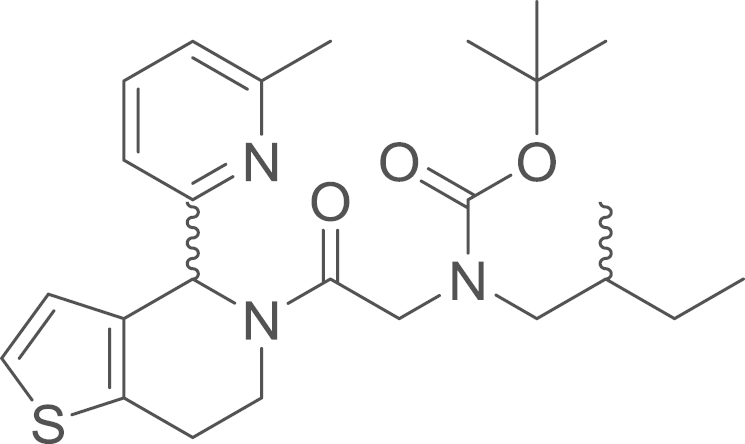

The amide (**8d**) was obtained from 4-(6-methyl-2-pyridyl)-1-thia-5-aza-4,5,6,7-tetrahydroindene (**4d**) (5.5 mg, 0.024 mmol, 1 eq) and [*N*-*tert*-Butoxycarbonyl(2-methylbutyl)amino]acetic acid (**7b**) (5.8 mg, 0.024 mmol, 1 eq) using general procedure K as yellow oil (9.8 mg, 0.021 mmol, 90%),^1^H NMR (400 MHz, CDCl_3_) δ=7.52 (q, ^*3*^*J*=7.0, 6.4 Hz, 4 H), 7.16 (d, ^*2*^*J*=4.3 Hz, 3 H), 7.09 (t, ^*3*^*J*=6.6 Hz, 4 H), 6.89 (dd, ^*3*^*J*=24.5, 5.1 Hz, 4 H), 6.58 (s, 1 H), 6.01 (d, ^*2*^*J*=17.2 Hz, 2 H), 4.94 (dd, ^*2*^*J*=24.0, ^*3*^*J*=9.4 Hz, 1 H), 4.74–4.53 (m, 2 H), 4.09–4.02 (m, 1 H), 3.45 (dd, ^*2*^*J*=14.9, ^*3*^*J*=7.2 Hz, 1 H), 3.33–3.17 (m, 1 H), 3.15–3.09 (m, 1 H), 3.06–2.80 (m, 6 H), 2.58 (s, 5 H), 1.48 (s, 15 H), 1.11 (ddd, ^*2*^*J*=18.7, ^*3*^*J* =13.3, 6.1 Hz, 4 H), 0.90 (m, 10 H).

### 2.5.4 2-(2-Methylbutylamino)-1-[4-(6-methyl-2-pyridyl)-1-thia-5-aza-4,5,6,7-tetrahydroinden-5-yl]-1-ethanone (9d)

**Figure:**
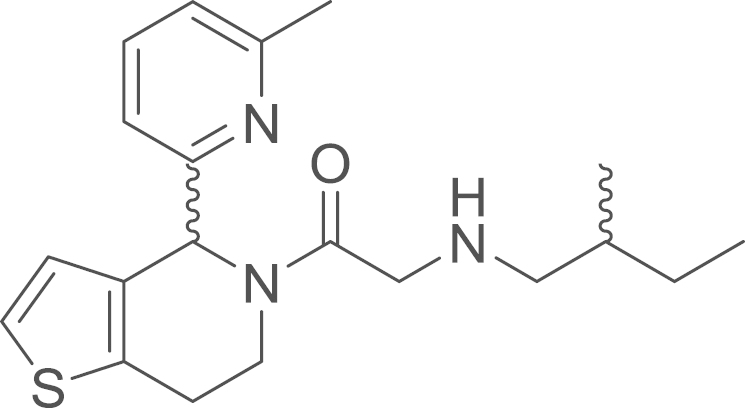

RU-SKI 201 (**9d**) was obtained from {2-[4-(6-methyl-2-pyridyl)-1-thia-5-aza-4,5,6,7-tetrahydroinden-5-yl]-2-oxoethyl}(2-methylbutyl)amino 2,2-dimethylpropionate (**8d**) (7.7 mg, 0.017 mmol, 1 eq) using general procedure L as yellow oil (1.9 mg, 0.0053 mmol, 31%). ^1^H NMR (400 MHz, CDCl_3_, rotameric ratio *E*:*Z* 7:3) δ=7.53–7.47 (m, 2 H), 7.15 (t, ^*3*^*J*=6.9 Hz, 1 H), 7.07 (t, ^*3*^*J*=7.5 Hz, 1 H), 6.99 (d, ^*3*^*J*=7.4 Hz, 1 H), 6.88 (d, ^*3*^*J*=5.2 Hz, 1 H), 6.85 (d, ^*3*^*J*=5.1 Hz, 1 H), 6.79 (d, ^*3*^*J*=7.7 Hz, 1 H), 6.56 (s, 1 H), 5.96 (s, 1 H), 4.96–4.87 (m, 1 H), 4.21 (d, ^*2*^*J*=16.3 Hz, 1 H), 4.09–3.95 (m, 1 H), 3.83 (dd, ^*2*^*J*=16.3, ^*3*^*J*=4.0 Hz, 1 H), 3.60 (qd, ^*2*^*J*=16.3, ^*3*^*J*=15.9, 5.3 Hz, 1 H), 3.06–2.83 (m, 4 H), 2.70–2.39 (m, 8 H), 1.70–1.39 (m, 3 H), 1.17 (dddd, ^*2*^*J*=23.2, ^*3*^*J*=20.5, 15.5, 7.6 Hz, 2 H), 0.97–0.85 (m, 10 H); ^13^C NMR (101 MHz, CDCl_3_) δ=170.22, 169.70, 159.07, 158.88, 158.60, 137.06, 136.77, 135.78, 134.25, 133.06, 132.13, 126.73, 126.35, 123.72, 123.00, 122.67, 122.07, 119.00, 118.53, 58.90, 56.69, 56.43, 56.19, 51.41, 50.97, 40.85, 37.04, 35.07, 34.55, 31.09, 27.48, 27.39, 25.78, 25.00, 24.82, 24.64, 17.75, 17.60, 11.35, 11.29; IR υ_MAX_ (neat)/cm^−1^: 3401.51 (br, C–H stretch, Ar–H), 3004.68, 2920.3 (CH_3_, -CH_2_-, alkyl), 1660.01 (C=O stretch, amide), 1436.89, 1406.87, 1314.26, 1261.75, 1015.25, 951.75, 900.43, 702.76, 670.50; HRMS (ESI, *m/z*): calcd. for C_20_H_28_N_3_OS^+^ [M+H]^+^, 358.1963; found, 358.1953 [M+H]^+^.

## 2.6 NMR spectra

### 2.6.1 RU-SKI 41 NMR spectral data

**Fig. 3:**
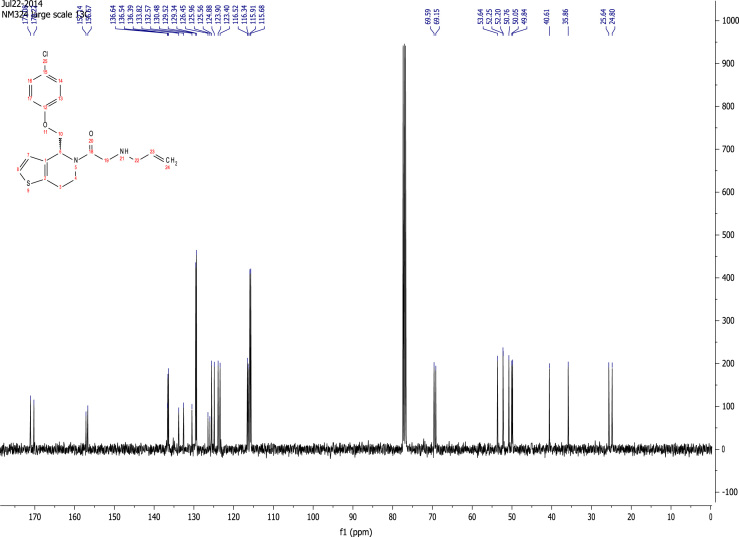
^13^C NMR (101 MHz, CDCl_3_) of RU-SKI 41 (**9a**).

**Fig. 4:**
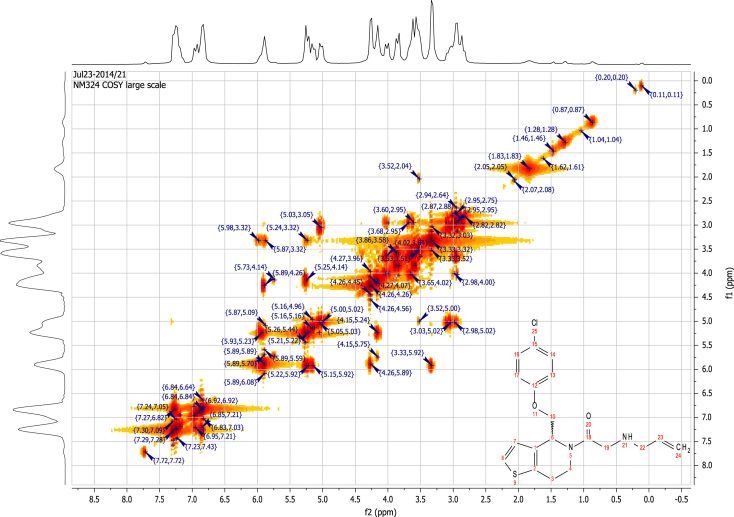
2D COSY (400 MHz, CDCl_3_) of RU-SKI 41 (**9a**).

**Fig. 5:**
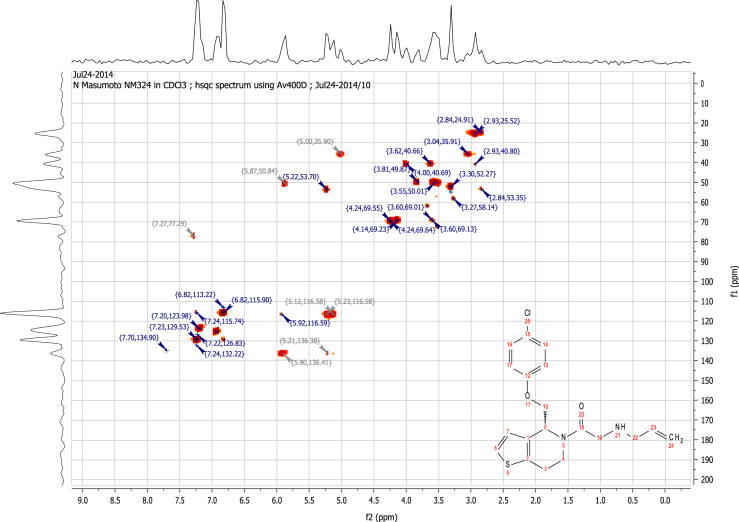
2D HSQC (400 MHz, CDCl_3_) of RU-SKI 41 (**9a**).

See Fig. 2, Fig. 3, Fig. 4, Fig. 5.

### 2.6.2 RU-SKI 43 NMR spectral data

**Fig. 6:**
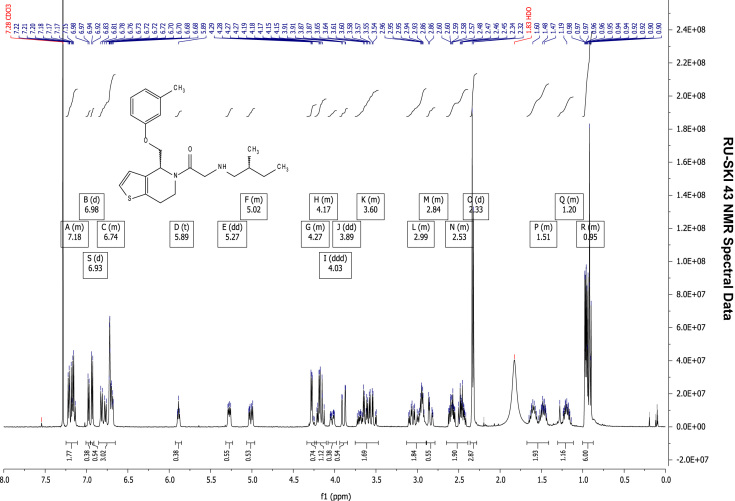
^1^H NMR (400 MHz, CDCl_3_) of RU-SKI 43 (**9b**).

**Fig. 7:**
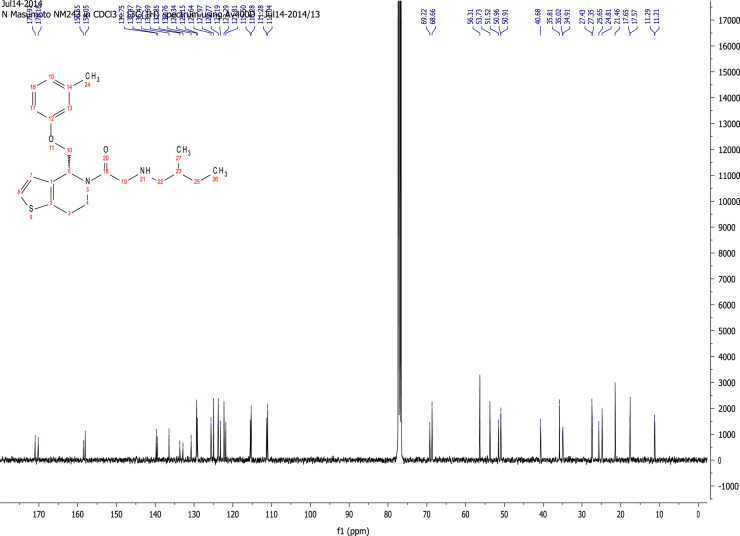
^13^C NMR (101 MHz, CDCl_3_) of RU-SKI 43 (**9b**).

**Fig. 8:**
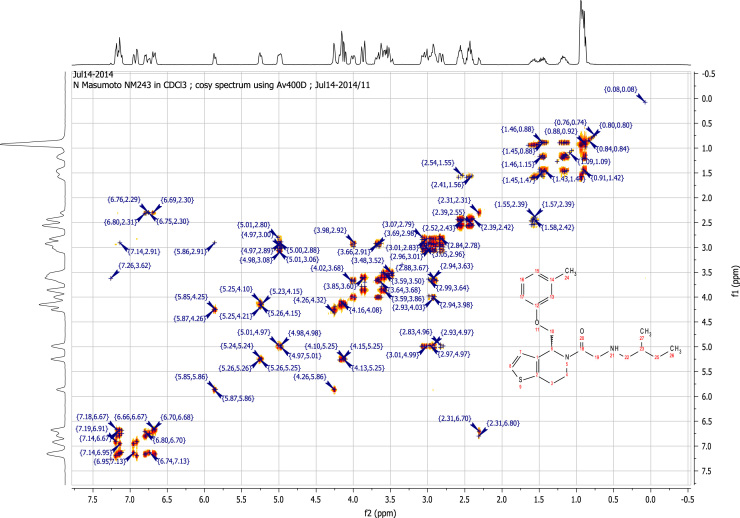
2D COSY (400 MHz, CDCl_3_) of RU-SKI 43 (**9b**).

**Fig. 9:**
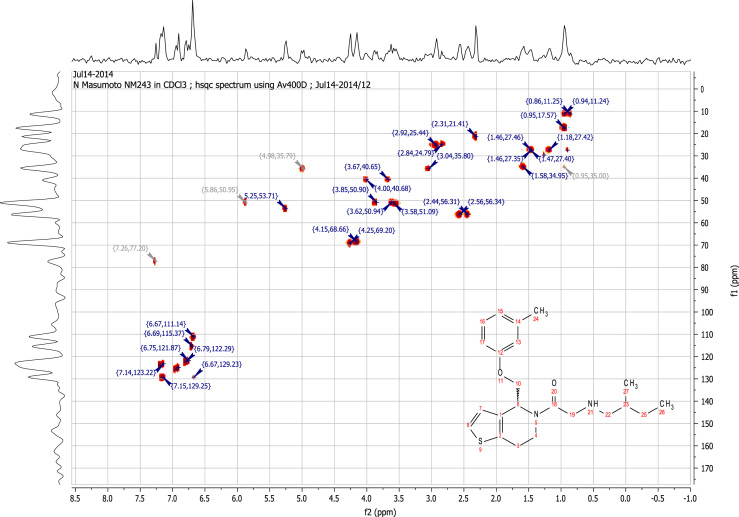
2D HSQC NMR (400 MHz, CDCl_3_) of RU-SKI 43 (**9b**).

See Fig. 6, Fig. 7, Fig. 8, Fig. 9.

### 2.6.3 RU-SKI 101 NMR spectral data

**Fig. 10:**
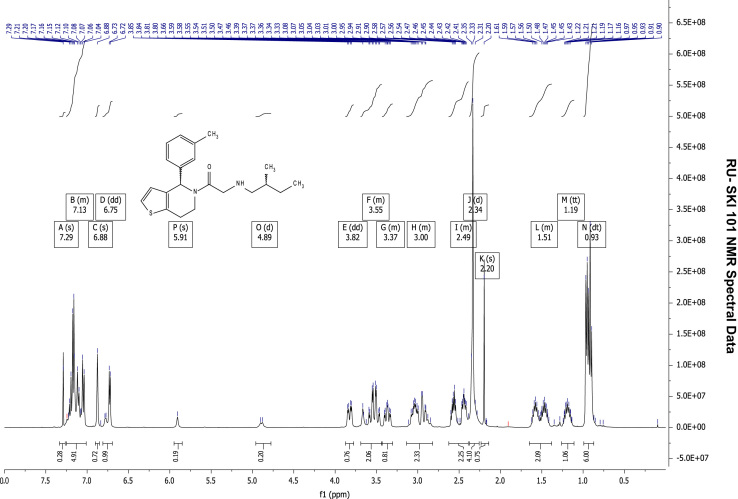
^1^H NMR (400 MHz, CDCl_3_) of RU-SKI 101 (**9c**).

**Fig. 11:**
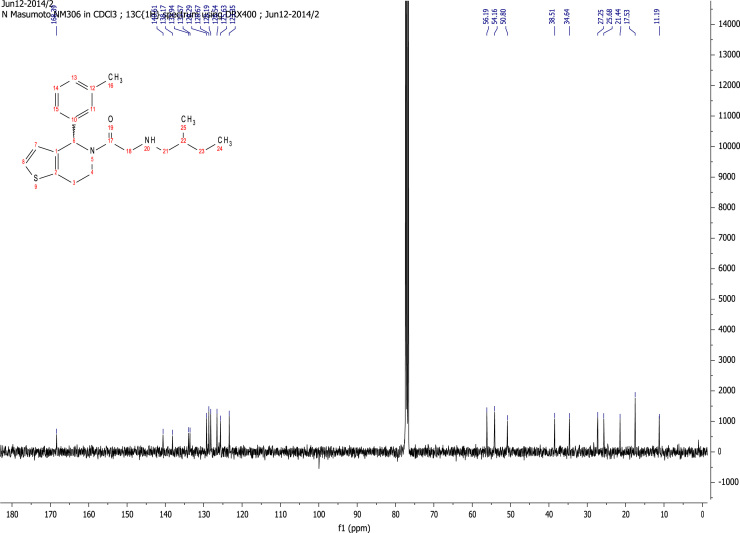
^13^C NMR (101 MHz, CDCl_3_) of RU-SKI 101 (**9c**).

**Fig. 12:**
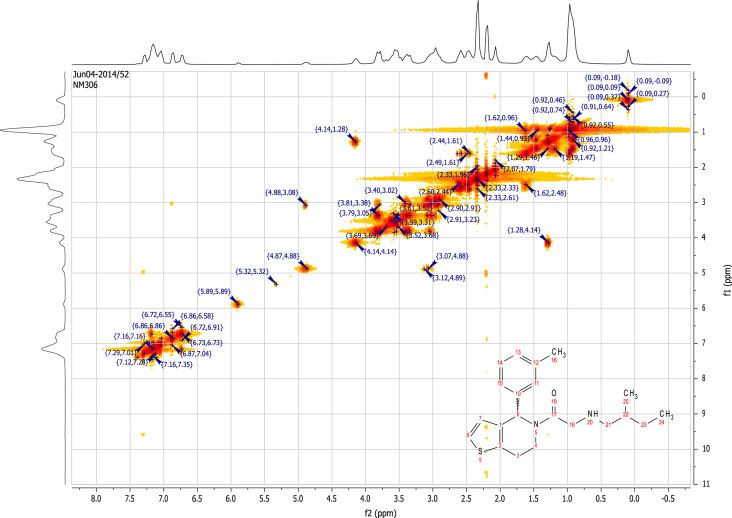
2D COSY (400 MHz, CDCl_3_) of RU-SKI 101 (**9c**).

**Fig. 13:**
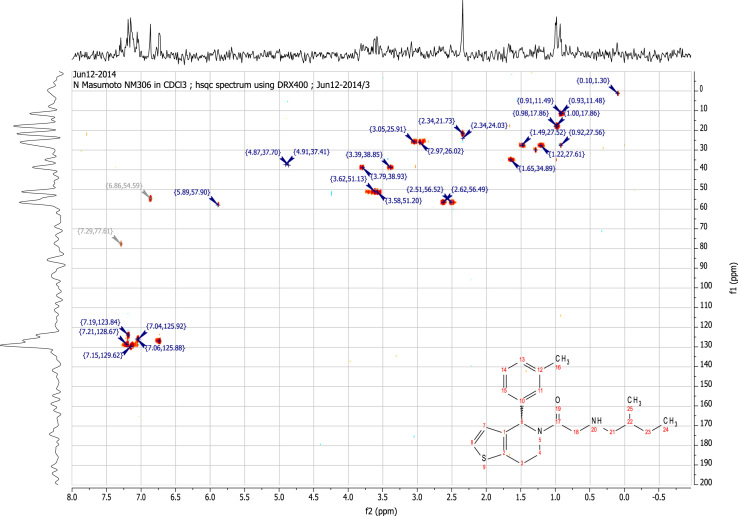
2D HSQC (400 MHz, CDCl_3_) of RU-SKI 101 (**9c**).

See Fig. 10, Fig. 11, Fig. 12, Fig. 13.

### 2.6.4 RU-SKI 201 NMR spectral data

**Fig. 14:**
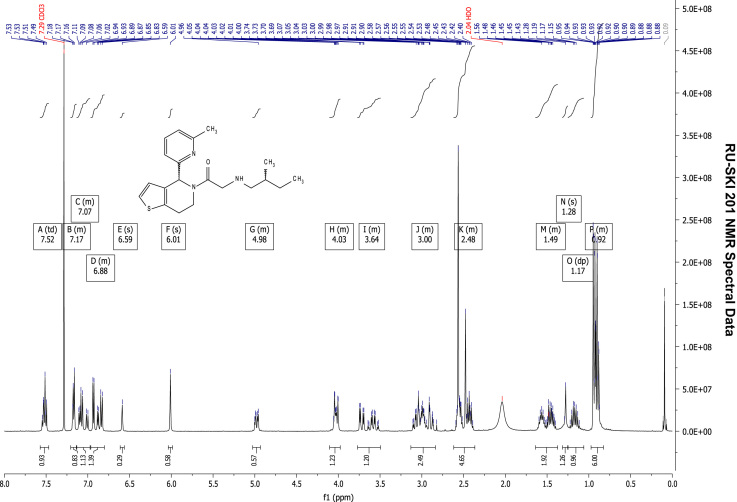
^1^H NMR (400 MHz, CDCl_3_) of RU-SKI 201 (**9d**).

**Fig. 15:**
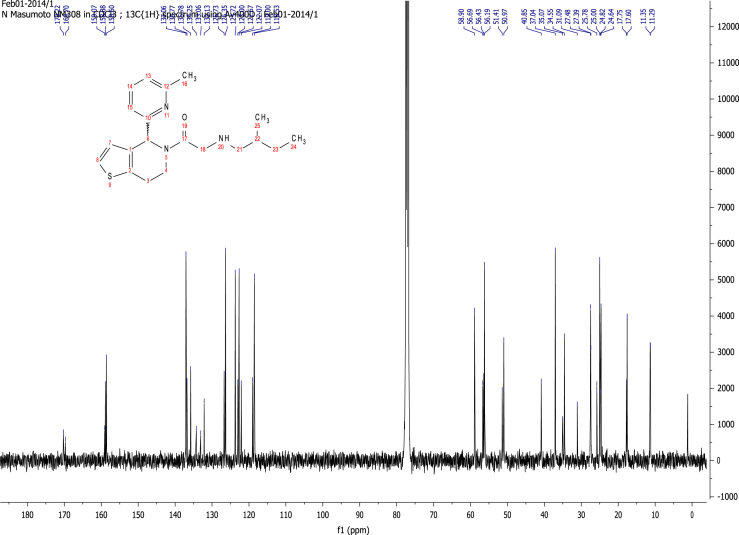
^13^C NMR (101 MHz, CDCl_3_) of RU-SKI 201 (**9d**).

**Fig. 16:**
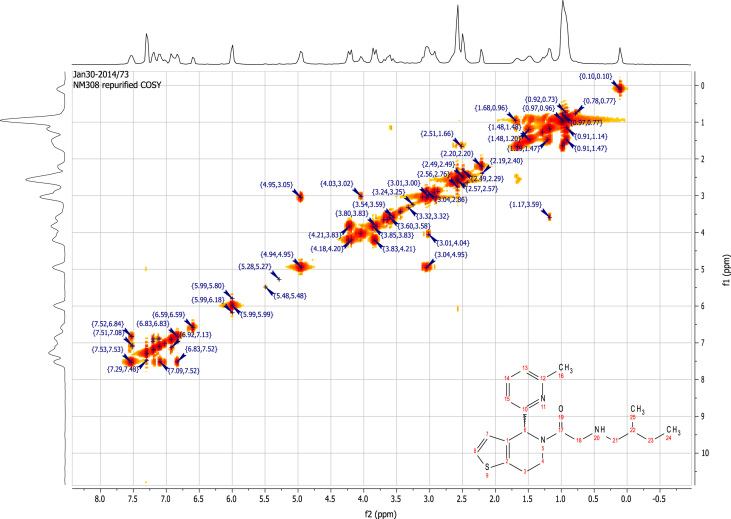
2D COSY (400 MHz, CDCl_3_) of RU-SKI 201 (**9d**).

**Fig. 17:**
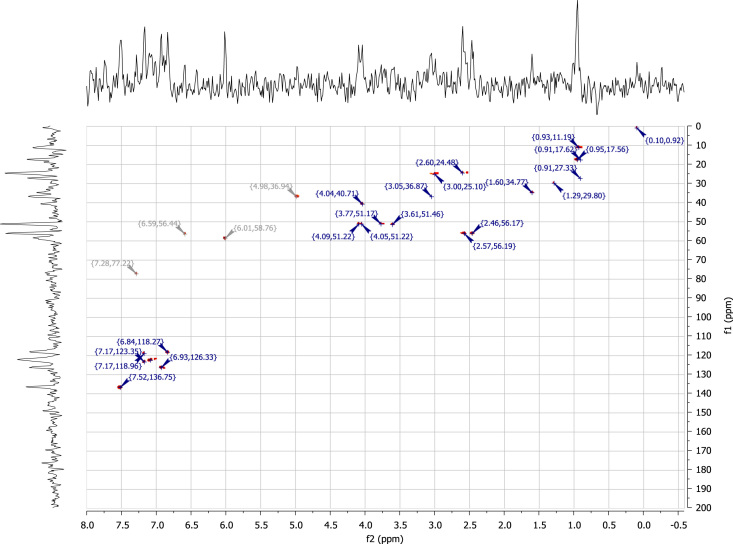
2D HSQC (400 MHz, CDCl_3_) of RU-SKI 201 (**9d**).

See Fig. 14, Fig. 15, Fig. 16, Fig. 17.

## Figures and Tables

**Table 1:** Amide conformational ratio data from RU-SKI inhibitors estimated by ^1^H NMR spectroscopy measured at 400 MHz in CDCl_3_ (Fig. 2, Fig. 6, Fig. 10, Fig. 14).

Compound	Observed *E*:*Z*
RU-SKI 41 (**9a**)	1:1
RU-SKI 43 (**9b**)	4:6
RU-SKI 101 (**9c**)	2:8
RU-SKI 201 (**9d**)	7:3
